# Characterization of the Glutathione *S*-Transferases Involved in Styrene Degradation in Gordonia rubripertincta CWB2

**DOI:** 10.1128/spectrum.00474-21

**Published:** 2021-07-28

**Authors:** Anna C. Lienkamp, Jan Burnik, Thomas Heine, Eckhard Hofmann, Dirk Tischler

**Affiliations:** a Microbial Biotechnology, Ruhr-Universität Bochum, Bochum, Germany; b X-Ray Structure Analysis of Proteins, Ruhr-Universität Bochum, Bochum, Germany; c Environmental Microbiology, TU Bergakademie Freiberg, Freiberg, Germany; University of Minnesota

**Keywords:** styrene metabolism, biotransformation, styrene oxide, glutathione, glutathione *S*-transferases, *Actinobacteria*, microbial ibuprofen production

## Abstract

The glutathione *S*-transferases carried on the plasmid for the styrene-specific degradation pathway in the *Actinobacterium* Gordonia rubripertincta CWB2 were heterologously expressed in Escherichia coli. Both enzymes were purified via affinity chromatography and subjected to activity investigations. StyI and StyJ displayed activity toward the commonly used glutathione *S*-transferase model substrate 1-chloro-2,4-dinitrobenzene (CDNB) with *K_m_* values of 0.0682 ± 0.0074 and 2.0281 ± 0.1301 mM and *V*_max_ values of 0.0158 ± 0.0002 and 0.348 ± 0.008 U mg^−1^ for StyI and StyJ, respectively. The conversion of the natural substrate styrene oxide to the intermediate (1-phenyl-2-hydroxyethyl)glutathione was detected for StyI with 48.3 ± 2.9 U mg^−1^. This elucidates one more step in the not yet fully resolved styrene-specific degradation pathway of Gordonia rubripertincta CWB2. A characterization of both purified enzymes adds more insight into the scarce research field of actinobacterial glutathione *S*-transferases. Moreover, a sequence and phylogenetic analysis puts both enzymes into a physiological and evolutionary context.

**IMPORTANCE** Styrene is a toxic compound that is used at a large scale by industry for plastic production. Bacterial degradation of styrene is a possibility for bioremediation and pollution prevention. Intermediates of styrene derivatives degraded in the styrene-specific pathways are precursors for valuable chemical compounds. The pathway in Gordonia rubripertincta CWB2 has proven to accept a broader substrate range than other bacterial styrene degraders. The enzymes characterized in this study, distinguish CWB2s pathway from other known styrene degradation routes and thus might be the main key for its ability to produce ibuprofen from the respective styrene derivative. A biotechnological utilization of this cascade could lead to efficient and sustainable production of drugs, flavors, and fragrances. Moreover, research on glutathione metabolism in *Actinobacteria* is rare. Here, a characterization of two glutathione *S*-transferases of actinobacterial origin is presented, and the utilization of glutathione in the metabolism of an *Actinobacterium* is proven.

## INTRODUCTION

Polystyrene is omnipresent in our daily lives in the form of diverse plastics and styrofoams. Its basic component is styrene, a volatile and toxic monoaromatic compound used at a large scale by industry (1–4). Currently, the demand for effective and standard waste disposal practices is increasing rapidly, especially in terms of sustainability. Styrene itself is toxic to most cell types (prokaryotes and eukaryotes) by interfering with cell membranes and other biomolecules, and, in addition, its metabolic activation by oxygenases leads to more problematic styrene oxide. The latter is true for prokaryotes and eukaryotes (3, 4). Despite a general toxicity of styrene for living organisms, some bacteria have evolved specific pathways for styrene degradation and thus can use it as a carbon source (3). These pathways are subject to research not only for bioremediation purposes but also for the generation of valuable compounds such as substituted phenylacetic acids, which can be generated in the course of these degradation routes (2–6). Until recently, one styrene-specific pathway was known besides some unspecific degradation routes (3). In the latter, styrene is cometabolized by enzymes, which typically act on other aromatics and their degradation intermediates. The central intermediates that are formed in these unspecific routes due to the relaxed substrate specificities are further converted by enzymes of general metabolism and enter the tricarboxylic acid (TCA) cycle (4). Hence, these nonspecific routes can lead to degradation or in other cases to biotransformation of respective compounds. In the known microbial styrene-specific degradation pathway, a monooxygenase converts styrene to its epoxide, which is further processed by an isomerase. Phenylacetic acid is then formed by a dehydrogenase (4). In 2018, it was first reported that a *Gordonia* strain carries a modified styrene-specific degradation route, which consists of elements found in the identified styrene-specific pathway and elements known for mammalian detoxification, including the activity of glutathione *S*-transferases (7). Those genes, including two putative glutathione *S*-transferases, were found to be significantly expressed only when styrene was supplied as a carbon source, determined by means of transcriptomic and proteomic approaches, respectively (7). Thus, a hybrid pathway for styrene was proposed, representing a 2nd styrene-specific degradative route. This discovery and the generally rare distribution of correlating enzymes among *Actinobacteria* convinced us to investigate this in more detail.

Glutathione *S*-transferases (GSTs; EC 2.5.1.18) belong to a ubiquitous protein superfamily (8–11). They catalyze the conjugation of the tripeptide glutathione (GSH) through a nucleophilic attack to an electrophilic group while having a broad substrate range (8). Their major role is cellular detoxification (9, 12, 13), and in bacteria, glutathione *S*-transferases contribute to the oxidative stress response and can be involved in basal metabolism (8). In *Actinobacteria*, the utilization of glutathione is rare, and the mainly featured low-molecular-weight thiol is mycothiol (MSH) (14–17). However, some *Actinobacteria* use an enzymatic set for the generation and utilization of glutathione via some metabolic pathways, which, so far known, mostly enable the accessibility of unusual carbon sources for the organism (17). Still, experimental proof for the utilization of glutathione *S*-transferases in the metabolism of *Actinobacteria* is scarce (14, 17). In *Rhodococcus* sp. AD45, a 300-kbp megaplasmid carrying the enzymatic set for isoprene degradation was identified (18, 19). Glutathione *S*-transferase activity has been proven to play an essential role in this degradative metabolic pathway (20), which was verified by a subsequent investigation of the two GSTs found in the organism’s genome, namely, IsoI and IsoJ (19, 21). And as mentioned above, later, a similar setup was discovered for the styrene-specific degradation pathway of another *Actinobacterium*, Gordonia rubripertincta CWB2, wherein the conversion of the epoxide also appears to be glutathione dependent (7). The respective genes are located on a 100-kbp plasmid, similar to the isoprene degradation cluster, which lead to assumptions about horizontal gene transfer (7, 18). Both pathways seem to have at least the first two enzymatic steps in common, specifically an epoxidation of the substrate via a monooxygenase and the subsequent opening of the epoxide ring through the conjugation of glutathione by a glutathione *S*-transferase (7, 19). Oelschlägel et al. (5) tested various styrene-degrading soil bacteria for the production of substituted phenylacetic acids. The generation of ibuprofen (4-isobutyl-α-methylphenylacetic acid) was only reported for a Gordonia rubripertincta CWB2 culture when fed with 4-isobutyl-α-methyl-styrene and styrene. Hence, this strain was suspected to differ significantly in its degradation pathway, because it somehow circumvents the narrow substrate range of the known styrene degraders. Later, its styrene monooxygenase gene was cloned and heterologously expressed, and the corresponding enzyme was investigated regarding a biocatalytic conversion of styrene and sulfides into chiral epoxide and sulfoxides (22). It converted styrene, with a specific activity of 0.42 U mg^−1^, predominantly to (*S*)-styrene oxide (>99% enantiomeric excess [ee]). This enzyme also accepted larger substrates, which is in agreement with the previously made observation that the styrene metabolic enzymes from strain CWB2 can handle larger substrates and thus lead to the generation of, for example, ibuprofen. Furthermore, this monooxygenase of strain CWB2 was phylogenetically analyzed and found to be most related to the subclade styrene monooxygenases of group E flavoprotein monooxygenases (22). But currently, the investigation of the pathway in Gordonia rubripertincta CWB2 is mostly restricted to the genetic level, supported by proteomics and transcriptomics, as well as to a single biocatalytic study of the first enzymatic step conducted by styrene monooxygenases (7, 22). However, the glutathione dependency of the epoxide opening was assayed in crude extract obtained from styrene-grown CWB2 biomass by monitoring the decrease of (*S*)-styrene oxide in the presence and absence of glutathione (7). Hence, the activity of a glutathione *S*-transferase was demonstrated for styrene-grown biomass for the first time, but only in crude preparation. This was confirmed by the already mentioned investigations of transcriptomics and proteomics, and the second styrene-specific degradation pathway was postulated (7). The genome of Gordonia rubripertincta CWB2 encodes adjacently for two proposed glutathione *S*-transferases designated StyI (NZ_CP022581.1, WP_119033946.1; 238 amino acids [aa]) and StyJ (NZ_CP022581.1, WP_119033945.1; 249 aa). These GSTs show an identity of 40% similarity on amino acid sequence for each other. The recent state of study did not clearly identify the active involvement of both GSTs or their specific activity in the styrene degradation pathway. Therefore, we aimed to clone both GSTs and conducted their heterologous expression in Escherichia coli with the possibility to purify them individually via affinity chromatography for a biochemical characterization. Both GSTs were characterized with the model substrate 1-chloro-2,4-dinitrobenzene (CDNB). Furthermore, activity toward styrene oxide was evaluated, and the postulated second enzymatic step of the degradation pathway was verified through the identification of the resulting glutathione conjugate.

## RESULTS

### Homology search and phylogenetic analysis.

**FIG 1:**
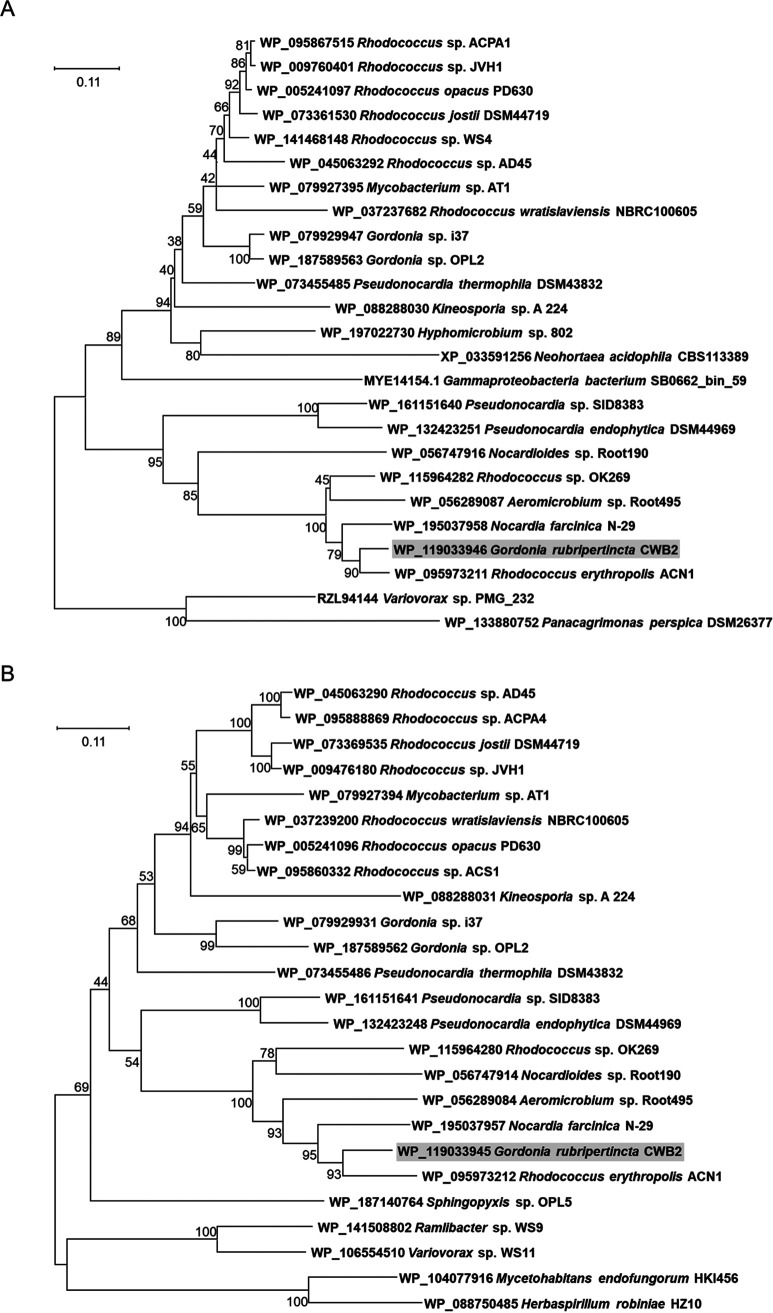
Phylogenetic trees of homologous sequences found via BLASTP in other organisms for the StyI (A) and StyJ (B) protein sequences. Both GSTs did not appear in the respective searches for another. The evolutionary history was inferred using the neighbor-joining method (52). The optimal tree is shown. The percentage of replicate trees in which the associated taxa clustered together in the bootstrap test (1,000 replicates) is shown next to the branches (47). The tree is drawn to scale, with branch lengths in the same units as those of the evolutionary distances used to infer the phylogenetic tree. The evolutionary distances were computed using the Jones-Taylor-Thornton (JTT) matrix-based method (48) and are in the units of the number of amino acid substitutions per site. This analysis involved 25 amino acid sequences. All ambiguous positions were removed for each sequence pair (pairwise deletion option). There were 250 (A) or 252 (B) positions in the final data set. Evolutionary analyses were conducted in MEGA X (49). Tree generation using the maximum likelihood or minimum evolution methods resulted in similar trees as the ones displayed.

Prior to experiments, an *in silico* analysis of the putative glutathione *S*-transferases found in Gordonia rubripertincta CWB2 was carried out to serve as preliminary placement in the superfamily of GSTs and to direct the planning of the experimental setup. BLASTP searches with the protein sequences of StyI (WP_119033946.1) and StyJ (WP_119033945.1) yielded different results. Though both do not seem to be homologous to each other, the hits with the highest similarity for both significantly appeared in the same organisms. A functional domain annotation with BLASTP also indicated different conserved functional regions, but each included a putative GSH-binding site and polypeptide-binding site (see Fig. S1 in the supplemental material). A potential dimer interface was only identified for StyJ. Phylogenetic analysis of 25 homologous GST sequences each is shown in Fig. 1. Homologous sequences for both StyI and StyJ are predominantly carried simultaneously by the same organisms, although not always. In this context, evolutionary distances also appear to be very similar. BLASTP searches for other enzymes known or suspected to take part in this styrene degradation pathway in the identified organisms revealed that each organism carries at least 5 related proteins (except *Gammaproteobacteria* bacterium SB0662_bin_59) (Table S2).

### General protein features.

**FIG 2:**
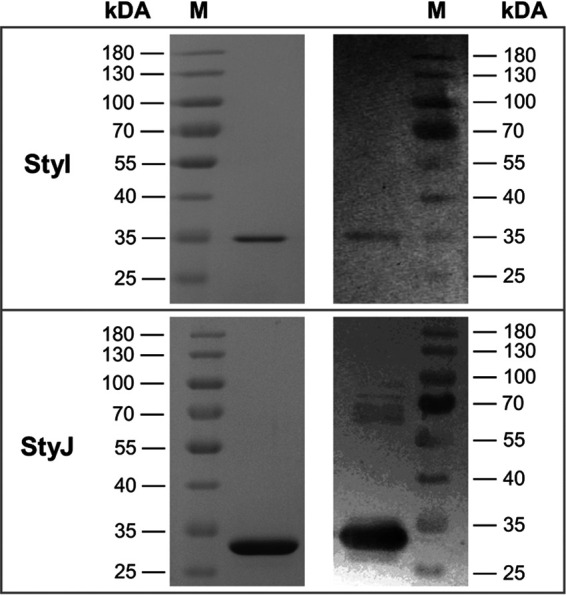
Protein staining pattern of SDS-PAGE (left) and immunodetection of a Western blot (right) of purified recombinant glutathione *S*-transferases StyI (estimated molecular weight from experiment was 35 kDa and theoretical molecular weight was 29.8 kDa) and StyJ (estimated molecular weight from experiment was 30 kDa and theoretical molecular weight was 30.1 kDa) from Gordonia rubripertincta CWB2.

Protein production and purification resulted in 47.3 ± 6.4 and 11.65 ± 1.8 mg of protein per liter of culture for StyI and StyJ, respectively (Table S1). Masses of purified recombinant GSTs were estimated by SDS-PAGE with approximately 35 kDa and 30 kDa for StyI and StyJ, respectively (Fig. 2). Calculated masses resulting from the amino acid sequences, including the His_10_ tag, were 29.8 kDa for StyI and 30.1 kDa for StyJ. Immunodetection of the His_10_ tag showed a faint signal for a protein of about 60 to 70 kDa for StyJ. This is assumed to be a dimer formed through a cysteine as a bridging group, which is presented shortly after the annotated dimer interface. Investigations of the thiol groups of both proteins were performed with the Ellman’s assay. The sequences of StyI and StyJ contain 1 and 4 cysteines in total, respectively. For purified StyI, 0.17 ± 0.01 thiol groups per protein monomer in a native state and 0.77 ± 0.1 thiol groups per protein monomer in an unfolded state were detected.

Thus, the cysteine of StyI is not exposed on the protein surface. Folded StyJ showed 1.42 ± 0.63 and unfolded StyJ showed 3.1 ± 0.07 thiol groups per protein. The addition of guanidine hydrochloride leads to protein unfolding but without reduction of disulfide bonds, reinforcing the assumption of a disulfide bridge between two StyJ units because its total cysteine content is 4 but only 3 were detectable per monomer. Of these, at least one seems to be a free thiol exposed on the protein surface. Size exclusion chromatography (SEC) of purified proteins resulted in one peak resembling 51 kDa for StyI and two peaks for StyJ, equaling 85 and 176 kDa (Fig. S3). The latter eluted close to the void volume and was out of the range of the calibration curve. Thus, the size cannot be determined reliably. However, elution close to the void volume indicates aggregation of the protein rather than oligomerization. With a measured native size of 51 kDa, StyI appears 1.5 times bigger than the estimated masses via SDS-PAGE (35 kDa). StyJ was estimated to be 30 kDa by SDS-PAGE and was measured with a 2.8 times-larger size of 85 kDa by SEC. Deviations in native sizes from the calculated molecular masses as well as the sizes detected by SDS-PAGE are most likely due to a different running behavior of the GSTs compared to the globular standard proteins used for calibration. However, the results also indicate for StyI a most likely monomeric to dimeric state and for StyJ a predominantly dimeric to trimeric state. SDS-PAGE and Western blotting of the collected peak fractions (Fig. S4) are equal to Fig. 2, ensuring the purity and identity of the samples. Respectively, the two proteins StyI and StyJ behave differently with respect to their hydrodynamic properties. A thermal shift assay of the transferases gave ambiguous results but showed a tendency for a melting point below 40°C (Fig. S2). Ultraviolet-visible (UV-Vis) spectra from 200 to 600 nm showed a single peak at 280 nm and no indication for any absorbing prosthetic groups.

### Activity of StyI and StyJ with model substrates.

**FIG 3:**
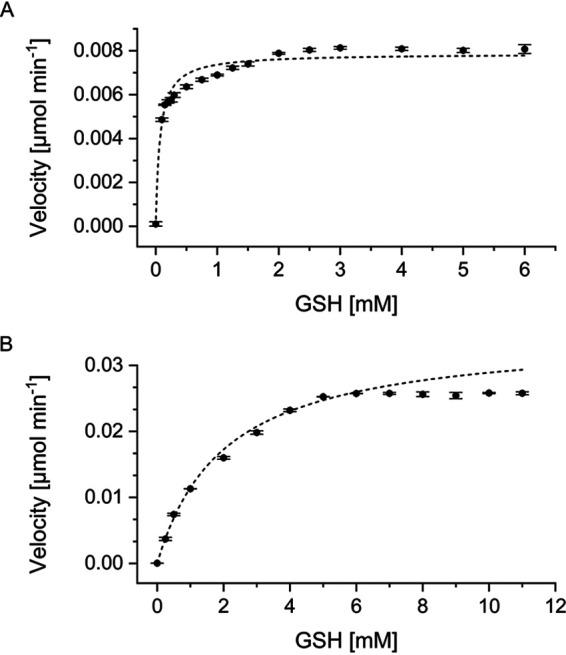
Michaelis-Menten kinetics determined for 1.5 mM CDNB and various concentrations of GSH. (A) StyI resulted in a *K_m_* value of 0.0682 ± 0.0074 mM and a *V*_max_ value of 0.0079 ± 0.0001 U (*R*^2^ = 0.9474); 0.5 mg ml^−1^ (16.8 μM) enzyme was applied. Enzyme-free background activity was 0.0031 ± 0 U with 6 mM GSH. (B) StyJ resulted in a *K_m_* value of 2.0281 ± 0.1301 mM and a *V*_max_ value of 0.0348 ± 0.0008 U (*R*^2^ = 0.9978); 0.1 mg ml^−1^ (3.3 μM) enzyme was applied. Enzyme-free background activity was 0.0037 ± 0.0002 U with 11 mM GSH.

Michaelis-Menten kinetics were determined with 1.5 mM CDNB while applying various concentrations of GSH. Calculations resulted in *K_m_* values of 0.0682 ± 0.0074 mM and 2.0281 ± 0.1301 mM and *V_max_* values of 0.0079 ± 0.0001 U and 0.0348 ± 0.0008 U for StyI and StyJ, respectively (Fig. 3). Data applied for calculation were not corrected for background activity for simplification purposes. Calculations with corrected data sets led to similar values. No activity could be measured for the other GST model substrates 1,2-dichloro-4-nitrobenzene (DCNB) and 4-nitrophenyl acetate (NPA). Incubation of different ratios of mixtures of StyI and StyJ showed no effect on the activity. At pH 6.5, both proteins were most stable in potassium and sodium phosphate buffer followed by bis(2-hydroxyethyl)iminotris(hydroxymethyl)-methane (BisTris)-HCl. StyJ precipitated quickly in 2-(*N*-morpholino)ethanesulfonic acid (MES) and 3-(*N*-morpholino)propanesulfonic acid (MOPS). StyI was still active in MES and precipitated directly in MOPS. StyI was most stable at increasing pH, showing highest activity at a storage pH of 8. It is stable up to 40°C, and the optimal temperature for activity was measured at 37°C. For StyJ, the optimal storage pH was 7 with less activity at a higher pH.

StyJ is temperature stable up to 35°C, with the highest activity at an optimal temperature of 30°C. Temperature stability and optimum temperature are in agreement with the tendency (below 40°C) given by the thermal shift assay for both enzymes as mentioned above. The incubation of both enzymes with different additives showed mixed effects, as shown in Table 1. Mentionable herein is the inhibition of StyJ by two-thirds in the presence of oxidized glutathione (GSSG). No additive gave an increased activity.

**TABLE 1:** Activity of StyI and StyJ when incubated with different additives

Additive^*a*^	StyI^*b*^ (% activity)	StyJ^*b*^ (% activity)
None	100 ± 2	100 ± 1
Mn^2+^	85 ± 1	83 ± 1
K^+^	93 ± 2	92 ± 2
Mg^2+^	77 ± 1	96 ± 5
Ca^2+^	105 ± 1	84 ± 2
Na^+^	106 ± 4	79 ± 3
EDTA	75 ± 6	94 ± 3
GSSG	89 ± 2	27 ± 2

a Zn^2+^ and Co^2+^ lead to direct precipitation of the proteins, and the background activity of Fe^2+^ with the substrate was too high to get reliable data.

b Measurements were done in 100 mM BisTris-HCl buffer, pH 6.5, and are given in percent compared to activity without any additive (StyI, 100% = 0.0119 ± 0.0003 U mg^−1^; StyJ, 100% = 0.1121 ± 0.0015 U mg^−1^).

### Activity of StyI and StyJ with styrene oxide.

**FIG 4:**
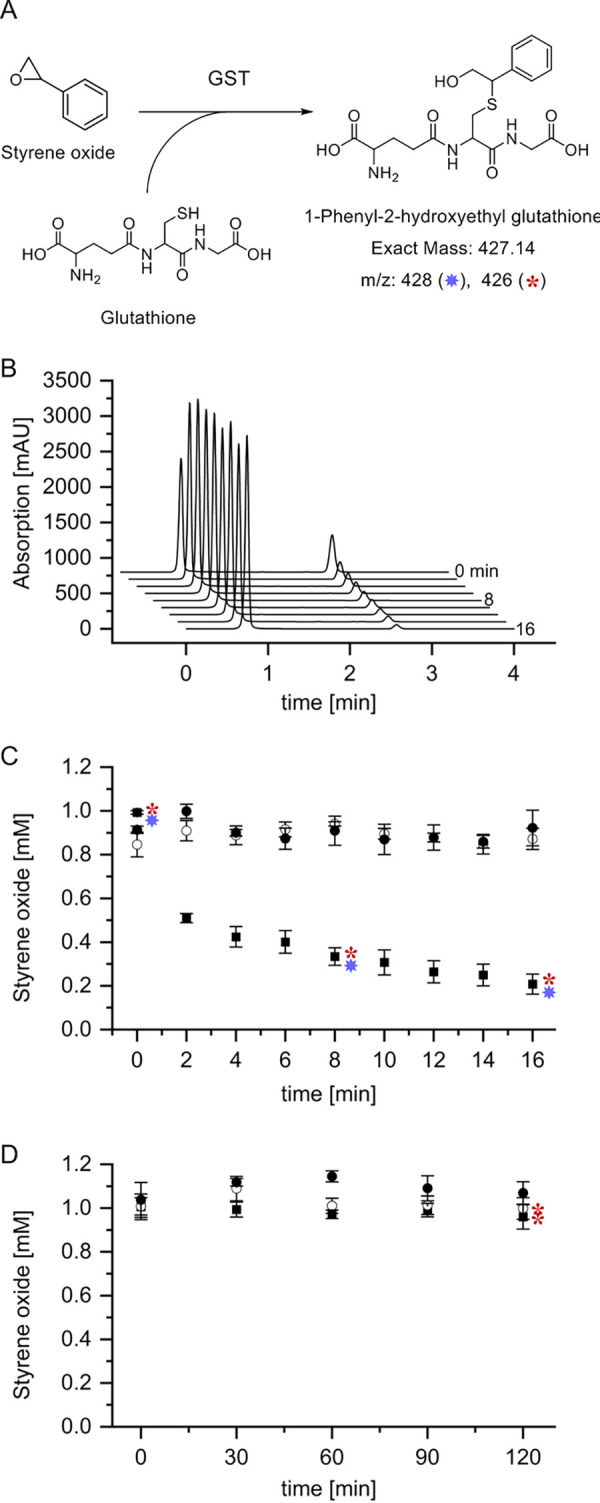
Degradation of styrene oxide by StyI and StyJ. (A) The reaction of styrene oxide with glutathione (GSH) catalyzed by a glutathione *S*-transferase (GST) as proposed for the styrene degradation pathway of Gordonia rubripertincta CWB2 by Heine et al. (7) would lead to the intermediate (1-phenyl-2-hydroxyethyl)glutathione (mass 427.14 g mol^−1^). (B) Stacked HPLC chromatograms for samples drawn at respective time points (0 to 16 min) for the degradation of styrene oxide (retention time of 2.6 min) by StyI. Samples were drawn from reaction mixtures containing 1 mM styrene oxide and 5 mM GSH over 16 min (5 μg ml^−1^ StyI) or 60 min (0.3 mg ml^−1^ StyJ) and analyzed via HPLC. (C and D) Degradation of styrene oxide by StyI (C) and StyJ (D) was detected via HPLC. A decrease in the concentration of styrene oxide was calculated by applying a recorded standard curve. Shown are the reactions (▪) and controls excluding either GSH (●) or the GST (○). LCMS analysis of selected HPLC samples showed corresponding *m*/*z* values, as assigned accordingly (428 in positive mode [*], 426 in negative mode [*]).

Glutathione-dependent degradation of styrene oxide could be measured for StyI with 48.3 ± 2.9 U mg^−1^. StyJ did not show any detectable activity despite increased incubation time and protein concentration. A mixture of StyI and StyJ (1:1) did not show an increase in activity. A corresponding *m*/*z* value for the intermediate, (1-phenyl-2-hydroxyethyl)glutathione with exact mass 427.14 g mol^−1^ as proposed by Heine et al. (7), was detectable by liquid chromatography mass spectrometry (LCMS) in positive mode (*m*/*z* of 428) and negative mode (*m*/*z* of 426) for reactions of StyI in all taken samples with increasing intensity. It was absent in all negative controls. After 2 h of incubation, an *m*/*z* value of 426 (negative mode) was detectable in StyJ samples and in the control excluding the GST at a very low intensity. This suggests an enzyme-independent reaction, which, in comparison to StyI, is significantly slower as the catalyzed reaction (Fig. 4). Hence, no enzyme-related conversion of styrene oxide was measured with StyJ.

## DISCUSSION

### Glutathione *S*-transferases StyI and StyJ in actinobacterial styrene degradation.

Glutathione *S*-transferases are ubiquitous enzymes that take part in a multitude of processes. They play an important role in cell detoxification by direct or indirect inactivation, degradation, or excretion of xenobiotics. Although the predominant thiol in *Actinobacteria* is mycothiol, some also utilize glutathione (16, 23). Thus, research on actinobacterial GSTs is rare (17). Two GSTs, IsoI and IsoJ, have been characterized in the isoprene degradation pathway of *Rhodococcus* sp. AD45 (19–21). Purified IsoI from isoprene-grown *Rhodococcus* sp. AD45 cultures was reported to conjugate isoprene monoxide to GSH, yielding 1-hydrox-2-glutathionyl-2-methyl-3-butene (HGMB) with an activity of 66 U mg^−1^ (21), while recombinant IsoI in E. coli crude extract assays showed an activity of 10 U mg^−1^ (19). It was not active with the GST model substrates CDNB or DCNB (19). On the other hand, E. coli crude extracts with recombinant IsoJ showed no conversion of isoprene monoxide but of the standard substrates CDNB and DCNB with 0.026 U mg^−1^ and 0.0064 U mg^−1^, respectively (19). Overall, this very much resembles the results for the homologous enzymes StyI and StyJ from Gordonia rubripertincta CWB2. StyI showed an activity of 48.3 ± 2.9 U mg^−1^ for the conjugation of GSH to styrene oxide, while for StyJ, no activity could be detected. StyJ (*V*_max_ = 0.0348 ± 0.0008 U; *K_m_* = 2.0281 ± 0.1301 mM) showed about 4 times more activity for the model substrate CDNB than StyI (*V*_max_ = 0.0079 ± 0.0001 U; *K_m_* = 0.0682 ± 0.0074 mM). It should be considered that the activity of StyI with the standard substrate CDNB was significant, but, with the sensitivity given by this method, the activity was too low to result in data points that enable a discrimination in the low-level range of the calculated *K_m_* and *V*_max_ values for StyI. This is not true for StyJ, for which reliable data points were obtained, and underlines the difference in substrate spectrum between both GSTs for epoxides or halogenated alkenes. Heine et al. (7) also detected a GSH-dependent degradation of (*S*)-styrene oxide in crude extracts of styrene-grown Gordonia rubripertincta CWB2 cultures with an activity of 44.23 U mg^−1^. Although this agrees with our results for StyI, the activities of a mixture of wild-type enzymes measured in crude extract with purified recombinant protein should not be compared due to the very different types of samples, as seen above with IsoI. Regarding other actinobacterial glutathione *S*-transferases, two GST isoenzymes were found in Streptomyces griseus with *K_m_* values of 0.25 ± 0.01 and 0.2 ± 0.01 mM for GSH measured with CDNB (24), but, due to the absence of GSH in this genus, a physiological function as a glutathione *S*-transferase is questionable (16). One bacterial GST from Escherichia coli was characterized with a *K_m_* value of 0.25 mM for GSH with CDNB (25). For phenol-degrading Pseudomonas strains, activities between 0.105 and 1.56 U mg^−1^ for CDNB, between 0.012 and 0.045 U mg^−1^ for DCNB, and between 0.063 and 0.269 U mg^−1^ for NPA were detected (26). GSTs are known to be a very diverse protein family and serve many different roles in different organisms, showing various functions, structures, activities, and substrate ranges (8). They appear as monomers, heterodimers, or homodimers (8, 9, 27). Here, StyI appeared as a monomer, while StyJ seems to form a homodimer. This is supported by the homology search, which predicted a dimer interface for StyJ. The appearance of respective bands on the Western blot suggested the formation of a bridging group, which is most likely an exposed cysteine close to the assigned dimer interface and was proven for StyJ through the Ellman’s assay. Possible formation of a heterodimer of StyI and StyJ was investigated through activity measurements after incubation of both purified enzymes. No increase in activity was detected. The overall data do not indicate the formation of a heterodimer as is known for other GSTs (27). It is still possible that a heterodimer is only formed through coexpression of both transferases, or other conditions are required for a successful oligomerization. Yet, the different substrate preferences regarding CDNB and styrene oxide as well as the alignment results suggest that StyI and StyJ play different roles in the metabolism of strain CWB2.

The protein superfamily of glutathione *S*-transferases currently consists of four different main classes, which evolved from at least three different protein folds, and are further divided into many subclasses (8, 14, 17). The results from the homology search suggest little homology for StyI and StyJ (Fig. 1; see also Fig. S1 in the supplemental material). StyI could not be directly related to a GST subgroup and IsoI-like transferases, as StyI seems to form a new class (17). StyJ was assigned as Ure2p-like in terms of GSH-binding site, dimer interface, and C-terminal polypeptide-binding site. Ure2 is a prion protein from yeast and shows significant similarities with beta-class GSTs (14). StyJ also shares conserved domains with the beta-class GstA from E. coli. Beta-class GSTs are the most common bacterial cytosolic GSTs and are limited to bacteria. They form homodimers and are able to conjugate CDNB (8, 14). A similarity to Ure2p-class transferases has also been recognized for IsoJ from *Rhodococcus* sp. AD45 (14). Two subclasses have been designated for Ure2p-class GSTs, named Ure2pA and Ure2pB. While the first is only found in fungi, Ure2pB-class GSTs have representatives in bacteria (28). Structures of two Ure2pB-class GSTs from E. coli revealed interesting differences as both have two binding sites for GSH. Moreover, YfcG (29) and YghU (30) show a higher affinity for GSSG (>100-fold) than for GSH or might bind two molecules of GSH simultaneously and exhibit an efficient disulfide bond oxidoreductase activity. Both form dimers and are active with CDNB (YghU, *K_m_* = 0.08 ± 0.02 mM for GSH). YghU showed inhibition through GSSG in the CDNB assay as observed for StyJ (Table 1). So far, inhibition of GSTs by oxidized glutathione is not known to be common. Although this effect was not relevant for the reactions assayed here because GSH remained conjugated to the substrates without generation of the disulfide, using this pathway for the generation of fine chemicals should be considered in the context of the overall cascade and, furthermore, for biotechnological applications. Another Ure2pB GST found in the fungus Phanerochaete chrysosporium (PcUre2pB1) is homologous to YfcG and YghU and is reported to deglutathionylate small molecules (28, 31). Only a few glutathione *S*-transferases are known to catalyze the reverse reaction, and most have been found in higher organisms, such as humans (32, 33), plants (34), and rats (35). Overall, it seems not unlikely that StyJ is an Ure2pB-class GST and, furthermore, serves in the styrene degradation pathway by removing the glutathione moiety later in the cascade. The isoprene degradation pathway in *Rhodococcus* sp. AD45 encloses two conversions of the glutathione conjugate through a dehydrogenase (IsoH) (21, 36). Further steps were not experimentally verified, but the generation of a CoA thioester through a CoA ligase was assumed followed by an IsoJ-catalyzed deglutathionylation to remove the GSH moiety through the generation of glutathione disulfide (19, 36).

**FIG 5:**
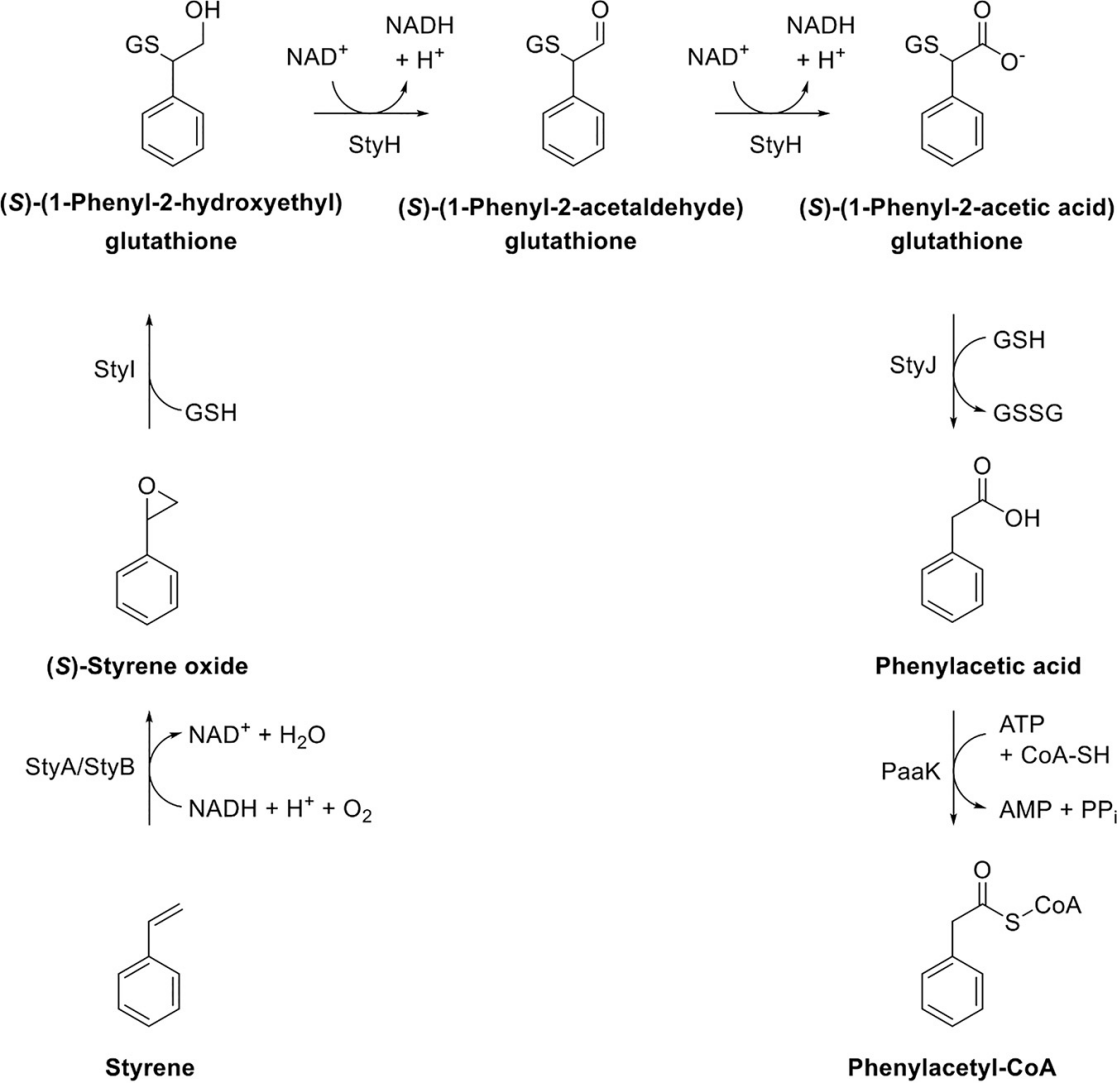
Styrene-specific degradation pathway in Gordonia rubripertincta CWB2 as proposed by Heine et al. and Lienkamp et al. (7, 17). The styrene monooxygenase StyA/StyB oxidizes styrene to styrene oxide. The opening of the epoxide ring through the conjugation of glutathione is catalyzed through the glutathione *S*-transferase StyI and generates the intermediate (1-phenyl-2-hydroxyethyl)glutathione. Conversions were experimentally measured. Enclosed conversions of StyH and a deglutathionylation by StyJ lead to phenylacetic acid, which is the central metabolite in the styrene-specific degradation pathway and is then funneled into the central metabolism via multiple enzymatic steps. PaaK, phenylacetate coenzyme A ligase.

StyJ displaying Ure2p-like features, as mentioned above, supports the hypothesis as made for the isoprene degradation pathway for the deglutathionylation of the intermediate; but, due to the generation of substituted phenylacetic acids solely through biotransformation with Gordonia rubripertincta CWB2 wild-type cultures as seen in Oelschlägel et al. (5), we would propose a different order of the reactions in CWB2 (Fig. 5), wherein the generation of phenylacetic acid through deglutathionylation comes prior to the formation of phenylacetyl-CoA, as already proposed by Heine et al. (7).

### Enzyme sets in detoxifying metabolism shared among *Actinobacteria* and relatives.

StyI and StyJ did not appear in the homology search for each other, but other homologs of these two unlike GSTs tend to occur together in different organisms (Fig. 1). The phylogenetic analysis shows a similar evolutionary relationship of the GST homologs for both GSTs. Heine et al. (7) already suggested horizontal gene transfer for the plasmid harboring the styrene degradation pathway in Gordonia rubripertincta CWB2. BLASTP searches for other proteins putatively taking part in this actinobacterial styrene degradation (StyABDGHIJ, Aldh1) as proposed by Heine et al. (7), for assumed GSH biosynthesis enzymes (GshA, GshB), as well as for homologs missing in CWB2 (StyCE) were done for the 32 organisms identified to carry homologs of StyI and StyJ. All organisms were found to carry different numbers and sets of these enzymes (see Table S2 in the supplementary material). When considering those organisms proven for styrene or isoprene degradation (12 in total) (5, 7, 20, 37–44), only one was found to harbor a StyC-like protein, which is supposed to be replaced by the GSTs in the styrene degradation route of CWB2. In total, three known degraders lack the GshA protein sequence. This can either suggest utilization of an alternative GSH biosynthesis protein for this step (17) or the utilization of another thiol. The only species described to degrade both styrene and isoprene is Rhodococcus opacus, for which no GshA homolog could be found. Overall, there is no bias for a certain combination of enzymes for isoprene or styrene degraders. Noteworthy might also be a tendency to lack the StyA homolog while still maintaining the degradation ability of some strains (4 out of 12). As evolution tends to eliminate obsolete protein sequences, especially in foreign plasmids, both GSTs seem to serve a certain function. For some organisms, the nature of the thiol or its biosynthesis appears more questionable.

### Conclusion.

The plasmid for styrene degradation in the *Actinobacterium* Gordonia rubripertincta CWB2 carries the genes for two glutathione *S*-transferases. StyI was proven to convert styrene oxide to the GSH conjugate (1-phenyl-2-hydroxyethyl)glutathione in the second step of the degradation cascade. This might explain the ability of CWB2, in contrast to other styrene degraders, to generate ibuprofen from 4-isobutyl-ɑ-methylstyrene (5). The role of StyJ in styrene metabolism remains controversial, although a deglutathionylation of the intermediate in a later step of the degradation route is plausible. Homologous proteins were found as sets in a number of *Actinobacteria* and *Proteobacteria* partly known to be isoprene or styrene degraders. This supports the hypothesis of horizontal gene transfer as well as a role of both GSTs in detoxification responses. StyI and StyJ originate from an interesting evolutionary background and offer new possibilities for biotechnological applications in cascade reactions for the production of drugs and other valuable compounds (3). StyI and StyJ are now two of five characterized actinobacterial glutathione *S*-transferases.

## MATERIAL AND METHODS

### Homology search and phylogenetic analysis.

A BLASTP (45) search on the nonredundant protein sequence database was performed with the native protein sequences of StyI (WP_119033946.1) and StyJ (WP_119033945.1). Multiple sequence alignments of nine closely related sequences each were generated using the online tool ClustalOmega (46) and the program GeneDoc (see Fig. S1 in the supplemental material). Furthermore, for each GST, 25 related glutathione *S*-transferases were chosen, and an alignment (ClustalW) was used to create distance trees via MEGA X using the maximum likelihood, minimum evolution, and neighbor-joining methods, each with a Jones-Taylor-Thornton (JTT) matrix-based model and 1,000 bootstrap replicates (47–52). Putatively involved proteins in styrene degradation in Gordonia rubripertincta CWB2 as identified by Heine (7) (StyABDGHIJ, Aldh1, and GshAB) were used to search for homologous proteins for similar degradation routes in the organisms identified during the tree generation. Proteins known to take part in styrene degradation in other *Actinobacteria* (Rhodococcus opacus 1CP and Pseudomonas sp. strain Y2) were also searched for homologs (StyCE) (7) in the above-mentioned organisms (Table S2).

### Chemicals and plasmids.

Styrene oxide (racemic), 1-chloro-2,4-dinitrobenzene (CDNB), 1,2-dichloro-4-nitrobenzene (DCNB), 4-nitrophenyl acetate (NPA), and 1-phenyl-1,2-ethanediol were purchased from Sigma-Aldrich (Steinheim, Germany). Reduced l-glutathione (GSH) and oxidized glutathione (GSSG) were ordered from Carl Roth (Karlsruhe, Germany).

The plasmids pET16bp_StyI and pET16bp_StyJ for the recombinant expression of the glutathione *S*-transferases carry the respective genes in a codon-optimized form synthesized by Eurofins MWG (NCBI GenBank accession numbers are MW590822 for *styI* and MW590823 for *styJ*). They were inserted into the multiple-cloning site (MCS) of pET16bp via the NdeI and NotI restriction sites, as described earlier for other genes (22). Correct insertion of constructs was checked via sequencing. Resulting expression products will carry an N-terminal His_10_ tag.

### Production of recombinant StyI and StyJ.

For protein production purposes, the expression strain E. coli BL21(DE3) was transformed with either pET16bp_StyI or pET16bp_StyJ for the heterologous expression of corresponding genes toward StyI or StyJ, respectively. Cultures were grown in Fernbach flasks in volumes of 1 liter (StyI) or 1.5 liters (StyJ) of LB medium with 100 μg ml^−1^ ampicillin at 37°C with shaking at 135 rpm. Induction was conducted at an optical density at 600 nm (OD_600_) of 0.4 to 0.6 by the addition of 0.1 mM isopropyl-β-d-thiogalactopyranoside (IPTG). Expression took place at 20°C with shaking at 135 rpm overnight. Cells were harvested by centrifugation (2,600 × *g*, 4°C, 45 min), washed once with buffer A (10 mM Tris-HCl, 500 mM NaCl, pH 7.5), and stored as a pellet at −20°C.

Cell disruption of fresh or thawed biomass was done in 20 ml of buffer A using a Bandelin Sonoplus (3 cycles, 1 min, 1-s pulse with a 1-s pause, 50% amplitude, tip MS72; Bandelin, Germany). Debris and soluble fractions were separated via centrifugation (17,500 × *g*, 45 min, 4°C). Affinity chromatography of His_10_-tagged proteins from the resulting soluble fraction was performed using 1-ml HisTrap HP columns with an ÄKTA Start system (GE Healthcare, Germany). Loading was done using buffer A, and a consecutive washing step with 5% buffer B (10 mM Tris-HCl, 500 mM NaCl, 500 mM imidazole, pH 7.5) removed nonspecifically bound proteins. Elution was performed using a linear gradient of 5 to 100% buffer B within 15 column volumes. Protein fractions were collected following the absorbance at 280 nm and subjected to dialysis at 4°C overnight. The final volume was decreased via centrifugal concentrators (Vivaspin turbo 15; 10,000 molecular weight cutoff [MWCO]; Sartorius, UK), and purified StyI or StyJ were either used directly or stored at −20°C. If necessary, buffers were exchanged afterwards either via PD-10 desalting columns (GE Healthcare, Germany) or Amicon Ultra-2 centrifugal filters (10-kDa nominal molecular weight limit [NMWL]; Sigma-Aldrich, Steinheim, Germany).

### Molecular mass estimation and protein quantification.

The molecular masses of the purified proteins in a denatured state were analyzed by SDS-PAGE (53, 54). Immunodetection of the His_10_-tagged proteins was performed via Western blotting (55) using a nitrocellulose blotting membrane (Amersham Protran 0.45-μm NC, GE Healthcare, Germany) and a Penta-His horseradish peroxidase (HRP) conjugate antibody (Qiagen, Germany).

Native sizes were analyzed via size exclusion chromatography on a HiLoad 16/600 Superdex 75 pg (GE Healthcare, Germany) using 10 mM Tris-HCl, pH 8, with 300 mM NaCl at a flow rate of 1 ml min^−1^ with an ÄKTA purifier (Amersham Pharmacia Biotech, UK). Calibration was performed with standard proteins of known sizes (RNase A, 13,700 Da; carbonic anhydrase, 29,000 Da; conalbumin, 75,000 Da; aldolase, 158,000 Da; blue dextran; Sigma-Aldrich, Steinheim, Germany) under similar conditions. The resulting standard curve was used for size determination of respective elution peaks of StyI and StyJ. Peak fractions were collected and analyzed by SDS-PAGE and Western blotting.

Final concentrations of pure proteins were calculated from the respective absorptions at 280 nm, applying molar extinction coefficients of 42,860 M^−1 ^cm^−1^ (StyI) and 48,150 M^−1 ^cm^−1^ (StyJ) as well as molecular weights of 29,836.3 g mol^−1^ (StyI) and 30,095.6 g mol^−1^ (StyJ) as predicted by Expasy ProtParam (56).

### Analysis of thiol groups and disulfide bonds.

The amount of surface and total thiol groups of the purified transferases was assayed by using the Ellman’s reagent 5,5′-dithio-bis(2-nitrobenzoic acid) (DTNB) (57). Its exposure to free thiol groups results in the formation of a mixed disulfide and 5-thio-2-nitrobenzoate ion (TNB^2−^), of which the latter can be detected photometrically at 412 nm. The detection of free surface thiol groups was conducted in 100 mM potassium phosphate buffer at pH 7.3 with 1 mM EDTA and 1 mM DTNB in a final volume of 1 ml. For the total thiol group content, 6 M guanidine hydrochloride was added to provoke enzyme unfolding. Per assay, 10 μM protein (0.3 mg ml^−1^) was applied, and the mixture was incubated for 30 min on ice prior to detection at 412 nm. Controls were performed without enzyme, and all measurements were done in triplicate. A standard curve was generated with various concentrations of l-cysteine (0 to 45 μM) applying the same conditions. The calculation of the thiol group concentration was done using either the standard curve or, alternatively, by applying the molar extinction coefficients of TNB^2−^ at 412 nm (14,150 M^−1 ^cm^−1^ in buffer, 13,700 M^−1 ^cm^−1^ in buffer with 6 M guanidine hydrochloride) (57, 58). Both resulted in very similar values; therefore, the data calculated with the standard curve are shown. The ratio of measured moles of thiol per applied moles of protein allowed an estimation of exposed thiol groups per molecule of protein (native and unfolded state).

### Thermal shift assay.

The thermal stability of the recombinant proteins was initially assayed using the thermal shift assay (59). Final protein concentrations of 4.5 μM mixed with the dye SYPRO orange (Thermo Fisher Scientific, Germany) were assayed in a CFX Connect Real-Time PCR detection system (Bio-Rad, Germany) using a temperature gradient of 20 to 90°C with an increase of 0.5°C per 10 s.

### Photometric enzyme assays.

Activity of glutathione *S*-transferases was assayed photometrically, as adapted from Habig and Jakoby (60). The standard assay was conducted in 100 mM potassium phosphate buffer at pH 6.5 in a final volume of 1 ml. Activity for StyI was determined using 5 mM GSH and 16.8 μM (0.5 mg ml^−1^) protein, while for StyJ, 9 mM GSH and 3.3 μM (0.1 mg ml^−1^) protein were applied. 1-Chloro-2,4-dinitrobenzene (CDNB) was added from an ethanol stock to a final concentration of 1.5 mM. The final concentration of ethanol was kept at 5% (vol/vol). Assay mixtures were preheated for at least 10 min, and reactions were started by addition of the enzyme. The conjugation of GSH to CDNB results in an absorption increase at 340 nm, which was monitored continuously for 2 min at 25°C in a Cary 60 spectrophotometer (Agilent, US) with an Alpha RA8 thermostat (LAUDA, Germany). Controls were measured by excluding the GST, GSH, or CDNB or replacing the enzyme with equimolar concentrations of bovine serum albumin (BSA). Measurements were done in triplicate if not stated otherwise. For calculation purposes, the nonenzymatic background (control excluding the GST) was subtracted from the reaction. A unit of enzyme activity was defined as the amount that catalyzes the formation of 1 μmol of substrate per min (μmol min^−1^). This was then used to calculate specific activities as unit per catalyst (U mg protein^−1^).

Kinetic parameters (*V*_max_ and *K_m_*) for glutathione-dependent reaction rates were determined with various concentrations of GSH (StyI, 0 to 6 mM; StyJ, 0 to 12 mM) and calculated via OriginPro2020 using the Levenberg Marquardt algorithm in the nonlinear curve fit mode. Values used for calculation of the kinetic parameters were not corrected for the nonenzymatic background reaction because a subtraction did not significantly affect the results. Respective background activity is stated in Results. 1,2-Dichloro-4-nitrobenzene (DCNB; 345 nm, 0.75 mM) and 4-nitrophenyl acetate (NPA; 400 nm, 1.5 mM) were tested as alternative GST model substrates in duplicate. Buffer feasibility at pH 6.5 was assayed with each 100 mM potassium and sodium phosphate buffer, 3-(*N*-morpholino)propanesulfonic acid (MOPS), 2-(*N*-morpholino)ethanesulfonic acid (MES), and bis(2-hydroxyethyl)iminotris(hydroxymethyl)-methane (BisTris) buffer. The effect of cations (Zn^2+^, Mn^2+^, K^+^, Mg^2+^, Co^2+^, Fe^2+^, Ca^2+^, and Na^+^ added as sulfates or chlorides), GSSG (oxidized glutathione), and EDTA on the enzymatic reaction was assayed under standard assay conditions in 100 mM BisTris-HCl buffer at pH 6.5. Enzymes were preincubated with 1 mM of the respective additive on ice for at least 10 min before addition to the assay containing a final concentration of 1 mM additive. The optimal temperature was assayed under standard assay conditions at different assay temperatures (20 to 40°C) starting the reaction with the substrate. For an investigation of temperature and pH stability, proteins were incubated for 10 min at different temperatures (20 to 50°C) or were rebuffered in 10 mM potassium phosphate buffer at various pH values (5.5 to 8) prior to addition to the standard assay. Enzymatic interaction of StyI and StyJ was tested by preincubation of mixed enzymes (500 μM StyI and 500 μM StyJ mixed in volume ratios of 1:1, 1:2, and 2:1) on ice. A final volume of 15 μl of enzyme mix was added to the standard assay containing 9 mM GSH, resulting in an overall protein concentration of 7.5 μM. For controls, one part of the mix was replaced by buffer (either StyI or StyJ). Due to the required amount of protein for all of the above-mentioned measurements, several batches of separately expressed and purified proteins were used (variation of activity between batches is displayed in Table S1).

### High-performance liquid chromatography analytics and glutathione conjugate detection by LCMS.

Enzyme activity toward styrene oxide was assayed in 50 mM potassium phosphate buffer at pH 7 with final concentrations of 5 mM GSH and 1 mM styrene oxide (racemic) in a total volume of 1 ml. The latter was provided from an acetonitrile stock while keeping the final acetonitrile concentration at 10%. The reaction was started by the addition of 5 μg ml^−1^ StyI or 0.3 mg ml^−1^ StyJ. Interaction of both was tested with a preincubated mix, resulting in final enzyme concentrations of 5 μg ml^−1^ each. The reaction was monitored at 25°C and 750 rpm over 16 min with sampling every 2 min for StyI and the mixture or over 120 min with sampling every 30 min for StyJ. The enzymatic reaction was quenched by mixing 1:1 with acetonitrile. Samples were centrifuged and subjected to high-performance liquid chromatography (HPLC) or LCMS analysis. Conversion of styrene oxide was monitored via reverse-phase (RP)-HPLC using a Eurospher C_18_ column (125 mm × 4 mm, 100-Å, 5-μm particle size; Knauer, Germany) with a flow rate of 1 ml min^−1^ and a mobile phase of 60% acetonitrile in water (isocratic mode). Commercial standards of styrene oxide (racemic), GSH, GSSG, and 1-phenyl-1,2-ethanediol were detected at 214 nm and resulted in retention times of 2.6, 0.7, 0.7, and 1.1 min, respectively. A calibration with defined concentrations of styrene oxide was used for quantification. The detection of the glutathione conjugate was performed on a UHPLCMS with electrospray ionization (ESI) using a hydrophilic interaction liquid chromatography (HILIC) column (Ascentis Express HILIC; 150 mm × 3 mm, 90-Å, 2.7-μm particle size; Sigma-Aldrich, Steinheim, Germany) with a mobile phase of 70% acetonitrile and 30% water (isocratic mode) and a flow rate of 0.3 ml min^−1^ for 10 min. Q1 and Q3 scans in positive and negative mode were conducted with a range of 100 to 600 *m*/*z*. All measurements were conducted in triplicate.
