# Supplementary material for: Characterization of the Glutathione S-Transferases Involved in Styrene Degradation in Gordonia rubripertincta CWB2
Source: Microbiol Spectr. 2021 Jul 28;9(1):10.1128/spectrum.00474-21. doi: 10.1128/spectrum.00474-21 (PMC8552685; doi:10.1128/spectrum.00474-21)
Supplement: SUPPLEMENTAL FILE 1 — Supplemental material. Download SPECTRUM00474-21_Supp_1_seq4.pdf, PDF file, 1.3 MB [file spectrum00474-21_supp_1_seq4.pdf]

Characterization of the glutathione *S*-transferases involved in styrene degradation in *Gordonia rubripertincta* CWB2

Anna C. Lienkamp[a#], Jan Burnik[c], Thomas Heine[b], Eckhard Hofmann[c], Dirk Tischler[a#]

[a]Microbial Biotechnology, Ruhr-Universität Bochum, Bochum, Germany
[b]Environmental Microbiology, TU Bergakademie Freiberg, Freiberg, Germany
[c]X-ray structure analysis of proteins, Ruhr-Universität Bochum, Bochum, Germany

[#]Correspondence to be addressed towards Anna C. Lienkamp (Anna.Lienkamp@rub.de) and Dirk Tischler (Dirk.Tischler@rub.de).

Supplemental Material

Alignment of homologous glutathione *S*-transferases. In a BlastP (1) search for sequences of StyI and StyJ (Accession no. WP_119033946.1 and WP_119033945.1, respectively) 8 homologous glutathione *S*-transferases were chosen for each sequence and two multiple sequence alignment were generated via ClustalOmega and GeneDoc (2) (Fig. S1). Functional domains as annotated by BlastP are indicated. StyI and StyJ do not display significant homology or similar domains for another.

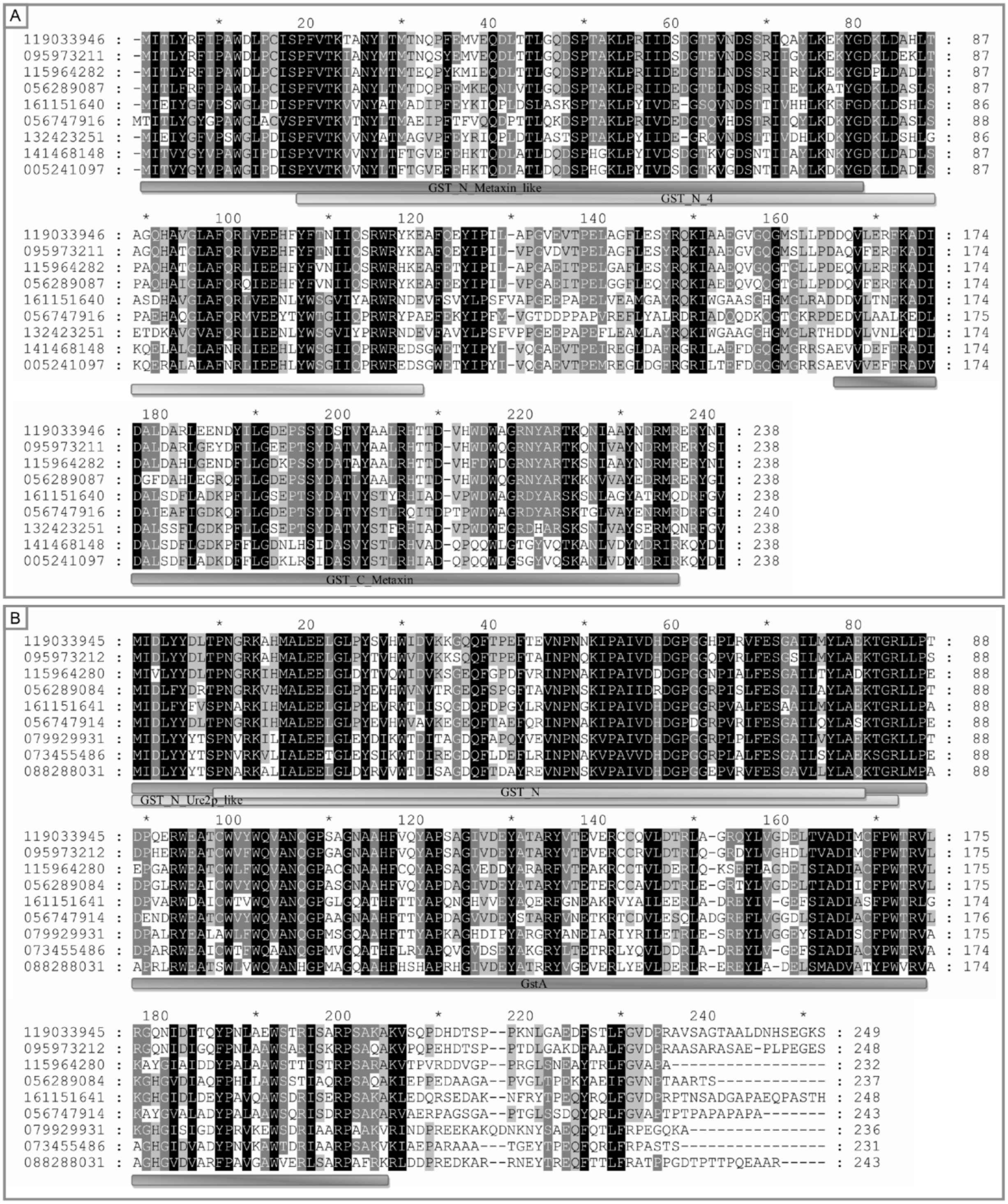
A
GST_N_Metaxin_like
GST_N_4
GST_C_Metaxin
B
GST_N
GST_N_Ure2p_like
GstA

Figure S1: Alignment of glutathione *S*-transferases. Sequences were chosen according to *e*-value and query coverage. The alignment highlights conserved residues. Conserved domains as identified by BlastP are annotated below the sequences. **A**: *Gordonia rubripertincta* (WP_119033946.1, **StyI**), *Rhodococcus erythropolis* (WP_095973211.1), *Rhodococcus* sp. OK269 (WP_115964282.1), *Aeromicrobium* sp. Root495 (WP_056289087.1), *Pseudonocardia* sp. SID8383 (WP_161151640.1), *Nocardioides* sp. Root190 (WP_056747916.1), *Pseudonocardia endophytica* (WP_132423251.1), *Rhodococcus* sp. WS4 (WP_141468148.1), *Rhodococcus opacus* (WP_005241097.1). Domains: GST_N_4 (pfam17172), GST_N_Metaxin_like (cd03080), GST_C_Metaxin (cd03193). **B**: *Gordonia rubripertincta* (WP_119033945.1, **StyJ**), *Rhodococcus erythropolis* (WP_095973212.1), *Rhodococcus* sp. OK269 (WP_115964280.1), *Aeromicrobium* sp. Root495 (WP_056289084.1), *Pseudonocardia* sp. SID8383 (WP_161151641.1), *Nocardioides* sp. Root190 (WP_056747914.1), *Gordonia* sp. i37 (WP_079929931.1), *Pseudonocardia thermophila* (WP_073455486.1), *Kineosporia* sp. A_224 (WP_088288031.1). Domains: GstA (COG0625), GST_N_Ure2p_like (cd03048), GST_N (pfam02798).

Thermal shift assay of StyI and StyJ. To assay the temperature stability of both glutathione *S*-transferases a thermal shift assay was conducted (3). The results were ambiguous but indicated melting points below 40 °C as shown in figure S2.

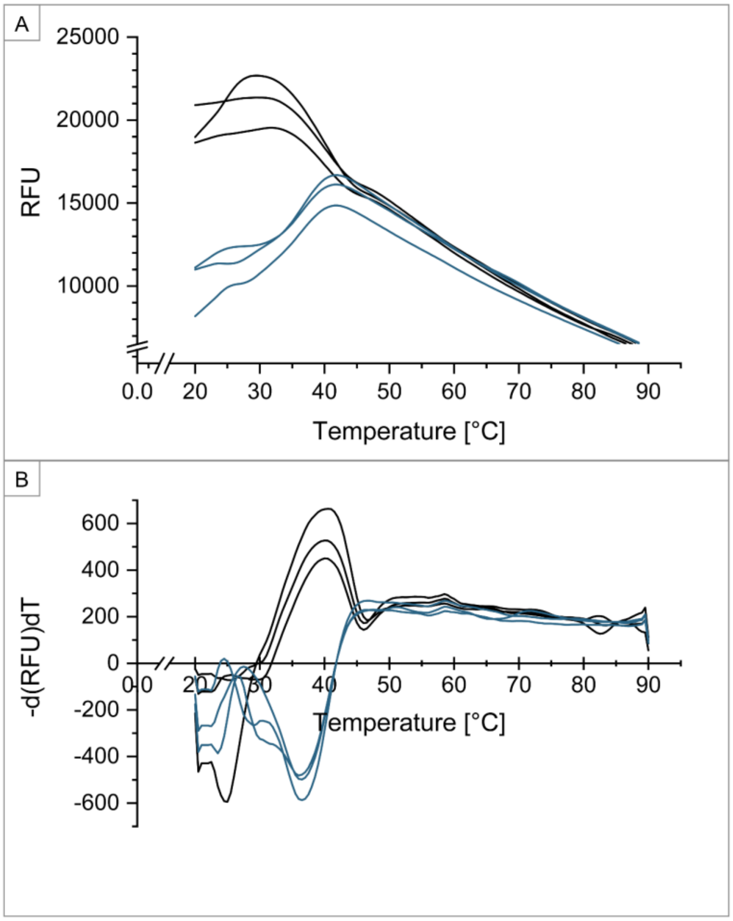

Figure S2: Thermal Shift Assay of StyI (black line —) and StyJ (blue line —). Final protein concentrations of 4.5 µM were analyzed using a temperature gradient of 20 to 90 °C with an increase of 0.5°C per 10 s. **A**: Melt curve. **B**: Melt Peak. Triplicates of StyI and StyJ are displayed.

Size exclusion chromatography of recombinant StyI and StyJ. Recombinantly produced enzymes were analyzed for their native size on a HiLoad 16/600 Superdex 75 pg (GE Healthcare, Germany) with an ÄKTA purifier (amersham pharmacia biotech, United Kingdom). Sizes were calculated applying measured calibration standards (Fig. S3). The collected peak fractions were checked *via* SDS-PAGE and western blot (Fig. S4).

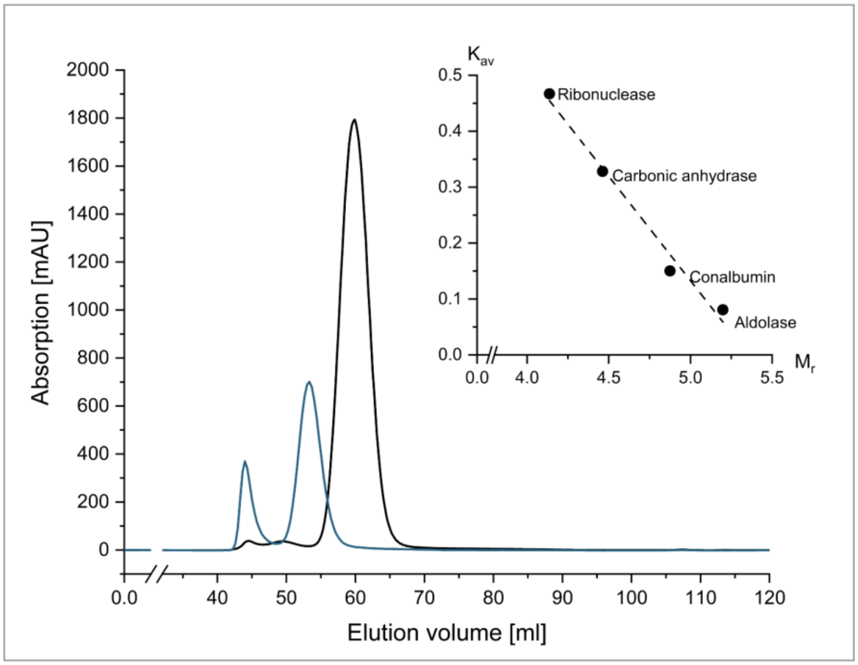

Figure S3: Size exclusion chromatography of StyI and StyJ with calibration curve. StyI (black line —) (59.9 ml, 51 kDa) and StyJ (blue line —) (53.3 ml 85 kDa and 44 ml 176 kDa). Calibration curve: Ribonuclease A (13700 Da, 77.8 ml), Carbonic Anhydrase (29000 Da, 66.8 ml), Conalbumin (75000 Da, 52.7 ml), Aldolase 158000 Da, 47.2 ml), Blue Dextran (void volume V_0=40.8 ml). Total column volume V_c=120 ml). R2=0.9835, equation: Y=-0.3728x+1.9967.

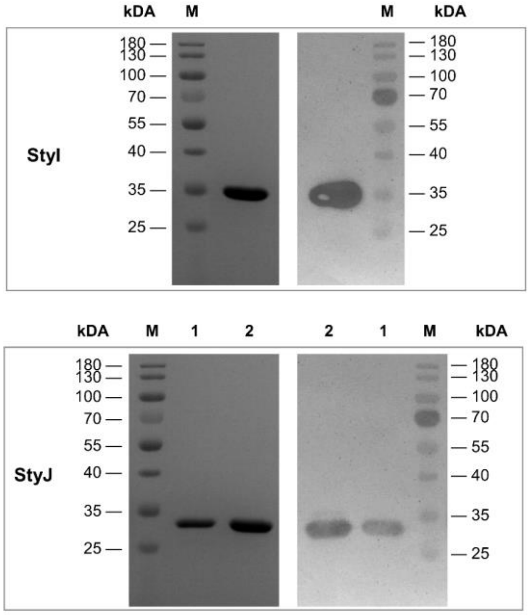

Figure S4: Protein staining pattern of SDS-PAGE (left) and immunodetection of western blot (right) analysis of gel filtration peaks of StyI and StyJ. StyI fraction at 59.9 ml. StyJ fractions at 44 ml (**1**) and 53.3 ml (**2**). Compare figure S3.

Activity of different purification batches. The amount of recombinant protein yielded per purification was not sufficient for all experiments. Therefore, multiple batches were purified for StyI or StyJ and used for respective assays. Batches were not pooled. Activities measured with the standard 1-chloro-2,4-dinitrobenzene (CDNB) assay are shown in table S1. In 100 mM potassium phosphate buffer pH 6.5 and a total volume of 1 ml the standard assay contained 1.5 mM CDNB. For StyI 5 mM GSH and 16.8 µM protein were used while for StyJ 9 mM GSH and 3.3 µM protein were applied. Measurements were conducted at 25°C monitoring the absorption increase at 340 nm for 2 min using a Cary 60 Spectrophotometer (Agilent, US).

Table S1: Activity of StyI and StyJ of different batches in U mg^{-1} and protein yields from respective expressions and purifications in mg protein per L culture. Standard conditions were used (except: **[A]** 9 mM GSH, 1.5 mM CDNB, 0.15 mg ml^{-1} StyI or 0.1 mg ml^{-1} StyJ and **[B]** 100 mM BisTris buffer pH 6.5).

StyI		StyJ	
[U mg^{-1}]	mg protein L culture^{-1}	[U mg^{-1}]	mg protein L culture^{-1}
0.0124±0.0020	48.2	0.1186±0.0095	10.4
0.0081±0.0003	55.5	0.2156±0.0041	-
0.0059±0.0002	-	0.1970±0.0135	-
0.0049±0.0004	-	0.2000±0.0163	-
0.0124±0.0011	45.6	0.1605±0.0036	12.9
0.0049±0.0001	40.0	0.0217±0.0005	-
0.0038±0.0001[A]	-	0.0146±0.0011[A]	-
0.0119±0.0003[B]	-	0.1121±0.0015[B]	-

References

1. Altschul SF, Gish W, Miller W, Myers EW, Lipman DJ. 1990. Basic local alignment search tool. J Mol Biol 215(3):403-410. https://doi.org/10.1016/S0022-2836(05)80360-2.
2. Madeira F, Park YM, Lee J, Buso N, Gur T, Madhusoodanan N, Basutkar P, Tivey ARN, Potter SC, Finn RD, Lopez R. 2019. The EMBL-EBI search and sequence analysis tools APIs in 2019. Nucleic Acids Res 47(W1):W636–W641. https://doi.org/10.1093/nar/gkz268.
3. Bai N, Roder H, Dickson A, Karanicolas J. 2019. Isothermal Analysis of ThermoFluor Data can readily provide Quantitative Binding Affinities. Sci Rep 9:2650. https://doi.org/10.1038/s41598-018-37072-x.
